# The AHL- and BDSF-Dependent Quorum Sensing Systems Control Specific and Overlapping Sets of Genes in *Burkholderia cenocepacia* H111

**DOI:** 10.1371/journal.pone.0049966

**Published:** 2012-11-20

**Authors:** Nadine Schmid, Gabriella Pessi, Yinyue Deng, Claudio Aguilar, Aurelien L. Carlier, Alexander Grunau, Ulrich Omasits, Lian-Hui Zhang, Christian H. Ahrens, Leo Eberl

**Affiliations:** 1 Department of Microbiology, University of Zurich, Zürich, Switzerland; 2 Institute of Molecular and Cell Biology, Singapore, Singapore; 3 Institute of Molecular Life Sciences, University of Zurich, Zürich, Switzerland; University Medical Center Utrecht, The Netherlands

## Abstract

Quorum sensing in *Burkholderia cenocepacia* H111 involves two signalling systems that depend on different signal molecules, namely *N*-acyl homoserine lactones (AHLs) and the diffusible signal factor *cis*-2-dodecenoic acid (BDSF). Previous studies have shown that AHLs and BDSF control similar phenotypic traits, including biofilm formation, proteolytic activity and pathogenicity. In this study we mapped the BDSF stimulon by RNA-Seq and shotgun proteomics analysis. We demonstrate that a set of the identified BDSF-regulated genes or proteins are also controlled by AHLs, suggesting that the two regulons partially overlap. The detailed analysis of two mutually regulated operons, one encoding three lectins and the other one encoding the large surface protein BapA and its type I secretion machinery, revealed that both AHLs and BDSF are required for full expression, suggesting that the two signalling systems operate in parallel. In accordance with this, we show that both AHLs and BDSF are required for biofilm formation and protease production.

## Introduction

Many bacteria are capable of coordinating gene expression in a cell density-dependent manner, a phenomenon commonly referred to as quorum sensing (QS) [1]. QS systems rely on the production and release of small signal molecules into the environment. Bacteria respond to these signals when their concentration has reached a certain threshold (and thus the bacterial population has attained a critical density), upon which expression of target genes is activated or repressed. Among the various QS signal molecules identified to date, the two most thoroughly investigated classes are the *N*-acyl-homoserine lactones (AHLs), which are produced by many Gram-negative bacteria, and small peptides, which are produced by many Gram-positive species [2], [3].

*Burkholderia cenocepacia* is a Gram-negative opportunistic pathogen belonging to the *Burkholderia cepacia* complex (Bcc), a group of 17 closely related bacterial species [4]. *B. cenocepacia* can cause airway infections in susceptible individuals, particularly in persons suffering from cystic fibrosis [5]. All members of the Bcc investigated so far utilize the AHL-dependent CepIR QS system [6]. CepI was shown to catalyze the synthesis of *N*-octanoyl homoserine lactone (C8-HSL) along with minor amounts of *N*-hexanoyl homoserine lactone (C6-HSL) [7]. At quorate population densities C8-HSL binds to its cognate transcriptional regulator CepR and in the AHL-bound form CepR binds to specific DNA sequences (so-called *cep* boxes) in the promoter region of target genes, thereby inducing or repressing gene expression [8]. Previous work has shown that the CepIR system regulates multiple functions, including virulence, biofilm formation, swarming motility, and the production of proteases, siderophores and antifungal compounds (reviewed in [9]). The CepR regulons of two *B. cenocepacia*, K56-2 and H111, have previously been determined using functional genomics approaches [8], [10], [11]. These investigations not only identified many genes encoding virulence factors [12] but has also shown that in strain H111 AHL-dependent expression of a large surface protein (*bapA*, BCAM2143) is critical for biofilm formation on abiotic surfaces [11].

Recent work has identified an additional QS system in *B. cenocepacia* that relies on BDSF (*Burkholderia* diffusible signal factor, *cis*-2-dodecenoic acid), which belongs to a rapidly growing family of fatty acid signal molecules [13], [14]. The biosynthesis of BDSF is driven by the product of *rpfF*_Bc_ (BCAM0581), an enoyl CoA hydratase [15]. RpfF_Bc_ is the first protein described to possess both dehydratase and thioesterase activity, which enables the direct conversion of the acyl carrier protein thioester of 3-hydroxydodecanoic acid into *cis*-2-dodecenoic acid [15]. More recently it has been demonstrated that the gene adjacent to *rpfF*_Bc_ encodes the BDSF receptor protein RpfR, which contains PAS-GGDEF-EAL domains [16]. It has been shown that upon binding of BDSF to RpfR the c-di-GMP phosphodiesterase activity of the protein is stimulated and as a consequence the intracellular c-di-GMP level is lowered. Hence, RpfR is the first example of a c-di-GMP metabolic enzyme that is directly activated by a cell-cell communication signal [16]. Disruption of either *rpfR* or *rpfF_Bc_* was shown to result in reduced motility, impaired biofilm formation, lowered proteolytic activity, and attenuated virulence [16]. All these phenotypes are also known to be AHL-regulated and we were therefore interested to investigate whether the two regulatory circuits regulate the same set of genes and whether they are interconnected or operate independently of each other.

In this study the BDSF stimulon of *B. cenocepacia* H111 was defined both at the transcript and protein level using RNA-Seq and shotgun proteomics. To determine the overlap of the AHL- and BDSF-dependent QS systems we compared the BDSF stimulon to the previously published CepR regulon [11]. In addition, we constructed a *cepI rpfF*_Bc_ double mutant and used it to assess the influence of the two signal molecules individually and in combination on biofilm formation and the production of proteases and on transcription of QS-regulated target genes. Our data demonstrate that, in spite of the observed decrease in AHL production in the *rpfF*_Bc_ mutant, the two QS systems regulate the tested phenotypes and genes independently, suggesting that they are not hierarchically arranged but operate in parallel under the experimental conditions used in this study.

## Results

### Mapping of the *B. cenocepacia* H111 BDSF Stimulon

**Figure 1:**
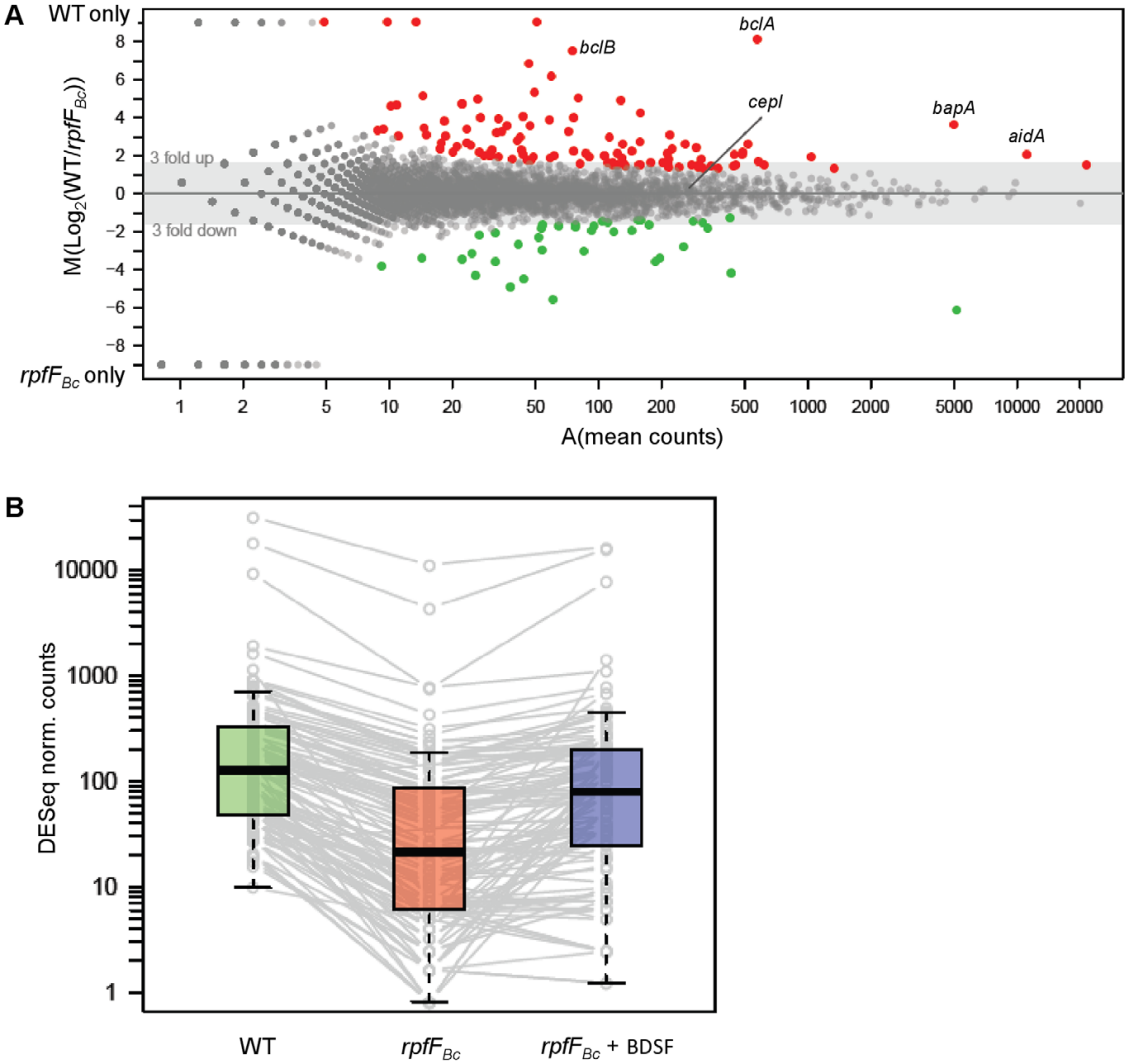
Mapping of the BDSF stimulon. (A) MA plot showing the fold change in transcript expression of all *B. cenocepacia* H111 genes versus the mean of identified reads in an *rpfF*_Bc_ mutant and wild type. The 112 down-regulated transcripts/proteins in the *rpfF*_Bc_ mutant are indicated in red, the 38 up-regulated genes in green. (B) Box plot demonstrating that supplementing the medium with 10 µM BDSF rescues the gene expression defects in the *rpfF*_Bc_ mutant.

To analyse the mode of action of the signalling molecule BDSF in strain H111, we conducted RNA-Seq genome-wide transcriptome analyses comparing the expression levels of a mutant in *rpfF*_Bc_ with those of the wild type and of the *rpfF*_Bc_ mutant supplemented by exogenous addition of BDSF. The sum of mapped reads matching to mRNA transcripts varied between 296’000–634’000 reads per sample. This yield of uniquely mapping reads is in the range of what Yoder-Himes and colleagues [17] have reported for RNA-Seq analyses of two *B. cenocepacia* strains [17]. Using the software DESeq [18], we focused on the 150 top ranked differentially expressed genes (Figure 1A), and noted that most of them (112 genes) were down-regulated in the *rpfF*_Bc_ mutant. For the majority of the down-regulated genes (85%), gene expression could be restored to wild type levels by supplementing the medium with 10 µM BDSF (Figure 1B). Among the 150 differentially expressed genes (Table S1), 38 showed increased expression in the *rpfF*_Bc_ mutant, suggesting that BDSF also represses a set of genes. For a functional analysis we transferred the classifications that were made by the EggNOG software [19] for *B. cenocepacia* strain J2315 to the respective orthologs in *B. cenocepacia* strain H111. Using the EggNOG resource, a large number of protein-coding genes could be functionally classified in 26 proNOG categories (5270, 73%, see Material and Methods). Many of the genes that showed decreased expression in the *rpfF*_Bc_ mutant were previously shown to be controlled by the CepI/CepR QS system, including genes encoding the sugar-binding lectins BclACB (BCAM0186-84), the large surface protein BapA (BCAM2143) and the protease ZmpB (BCAM2307). Additionally, two gene clusters coding for the exopolysaccharide cepacian (BCAM1004-10 and BCAM0854-0864) were identified. We next compared the *rpfF*_Bc_ regulon of strain H111 with the recently published BDSF regulon of strain J2315 [20]. Surprisingly, from the set of 150 top ranked genes identified as BDSF-regulated in our study only three genes overlapped with the BDSF regulon determined for strain J2315, namely a dioxygenase (BCAM0811), a component of a type I secretion system located downstream of *bapA* (BCAM2142) and the permease of an ABC efflux pump (BCAM2225). This discrepancy may be due to differences between the strains and culture conditions.

### Comparative Proteome Analysis of the *B. cenocepacia* H111 Wild Type and its Isogenic *rpfF_Bc_* Mutant Derivative

In order to obtain a comprehensive view of the RpfF_Bc_ regulon, we also analyzed the protein expression profiles of the wild type, the *rpfF_Bc_* mutant and the *rpfF_Bc_* mutant supplemented with BDSF. For this purpose, proteins and total RNA (used for the RNA-Seq study, see above) were extracted from each sample in parallel, allowing for the best possible comparison of these datasets. Using stringent criteria (see Material & Methods for details), a total of 2′565, 2′420, and 2′685 proteins were detected in wild type, *rpfF_Bc_* mutant and supplemented *rpfF_Bc_* mutant, respectively. Therefore, approximately 35% of the *in silico* predicted *B. cenocepacia* H111 proteins were identified. Based on the target-decoy database search results, we could estimate that the overall peptide and protein false discovery rate (FDR) amounted to 0.8% and 2.7%, respectively. Similar to reads from RNA-Seq, the individual peptide spectrum matches (PSMs) also represent count data that can be analyzed with the DESeq software to identify differentially expressed proteins. In total, 116 proteins displayed significant changes in the *rpfF_Bc_* mutant (Table S2). Out of these, 81 proteins were significantly down-regulated in the mutant. When the medium was supplemented with BDSF, protein expression was restored to wild type levels in 91.5% of the cases. A correlation between proteomic and transcriptomic data showed that 29 genes/proteins are shared among the top ranked candidates when combining the results from both approaches (Table 1).

**Table 1:** List of genes and proteins differentially expressed using RNA-Seq and proteomics in the *rpfF_Bc_* mutant compared to the wild type.

				RNA Seq		Proteomics	
Locus ID CCE^a^	Orthologues J2315^b^	Description^c^	Gene name	wt vs *rpfF* d	wt vs *rpfF* compl^e^	wt vs *rpfF* f	wt vs *rpfF* compl^g^
CCE49245	BCAL0111	O-linked N-acetylglucosamine transferase		3.0	2.1	na	2.8
CCE46674	BCAL0249	50S ribosomal protein L6		0.4	0.9	1.6	1.5
CCE48190	BCAL0524	Flagellar motor switch protein		5.1	3.2	3.9	1.4
CCE48189	BCAL0525	Flagellar MS-ring protein		4.2	6.5	2.9	1.6
CCE53354	BCAL0762	Methyl-Apting chemotaxis protein		4.6	1.4	2.7	0.9
CCE53285	BCAL0831	Phasin family protein		2.9	0.7	3.2	1.4
CCE51557	BCAL1059	Bifunctional N-succinyldiaminopimelate-aminotransferase/acetylornithine transaminase protein		2.8	1.6	0.5	0.8
CCE50475	BCAL2352	Carbonic anhydrase		4.3	1.0	7.0	1.1
CCE49840	BCAL3041	Protein involved in carbohydrate transport		4.5	2.0	0.5	1.6
CCE51244	BCAL3285	Nitric oxide dioxygenase; flavohemoprotein		6.7	0.6	na	0.8
CCE46722	BCAM0184	Fucose-Binding lectin protein	*bclB*	183.0	0.5	na	0.8
CCE46720	BCAM0186	Fucose-Binding lectin protein	*bclA*	280.2	0.5	na	1.1
CCE46715	BCAM0191	Non-ribosomal peptide synthetase		5.2	0.9	na	0.7
CCE46713	BCAM0192			9.4	1.5	na	1.2
CCE46711	BCAM0194			7.9	1.6	na	0.4
CCE46710	BCAM0195	Non-ribosomal peptide synthetase		6.6	1.4	na	0.8
CCE46709	BCAM0196			5.9	0.9	14.0	0.9
CCE50899	BCAM0853	Transposase		3.1	0.8	10.0	0.7
CCE50898	BCAM0854	Mannose-6-phosphate isomerase		8.3	0.4	na	0.4
CCE50892	BCAM0859	Protein involved in capsule organization		31.8	0.4	na	0.7
CCE48736	BCAM1004	Gdp-Mannose 4,6-dehydratase		4.3	0.3	na	0.5
CCE47169	BCAM1572	Methyl-Apting chemotaxis protein		3.0	1.2	3.0	1.5
CCE46959	BCAM1745	Atpase		0.01	0.8	0.2	2.2
CCE53120	BCAM2140	Type I secretion membrane fusion protein		15.8	1.0	na	1.4
CCE53119	BCAM2141	ABC transporter protein		16.0	0.9	na	1.0
CCE53117	BCAM2143	Calcium ion binding protein	*bapA*	12.2	0.8	41.5	0.7
CCE53024	BCAM2224	Protein involved in siderophore transport		31.5	28.0	na	na
CCE53019	BCAM2230	Non-ribosomal peptide synthetase		11.8	7.9	na	na
CCE46201	BCAM2627	Protein involved in iron ion transport		6.2	11.0	10.0	10.0

a Nomenclature according to GenBank file CAFQ01000001.1.

b Orthologs were identified as described in the Material and Methods section.

c Description according to the EggNOG classification.

d Fold change (FC) of transcript expression, comparing wild type strain with *rpfF* mutant grown in LB medium until an OD of 2.

e Fold change (FC) of transcript expression, comparing wild type strain with complemented *rpfF* mutant grown in LB medium until an OD of 2.

f Fold change (FC) of protein expression, comparing wild type strain with *rpfF* mutant grown in LB medium until an OD of 2.

g Fold change (FC) of protein expression, comparing wild type strain with complemented *rpfF* mutant grown in LB medium until an OD of 2.

na, not applicable because the read number or spectral counts in the mutant or complemented strains is equal 0.

### Comparison of the *B. cenocepacia* H111 RpfF_Bc_ and CepR Regulons

A recent transcriptomic study using a custom *B. cenocepacia* oligonucleotide microarray showed that expression of 103 genes was decreased at least two-fold in a *cepR* mutant derived from *B. cenocepacia* strain H111 [11]. When those data were compared to the RNA-Seq data obtained in the present study, a total of 31 overlapping genes were found to be differentially expressed in both the *cepR* and the *rpfF*_Bc_ mutant (Figure S1, Table 2). Among the co-regulated genes was the *bclACB* lectin operon and genes coding for the large surface protein BapA and enzymes for the biosynthesis of the exopolysaccharide cepacian (Table 2). Interestingly, expression of *aidA* and *cepI,* which were previously demonstrated to be directly controlled by CepR through binding of the CepR/C8-HSL complex to the promoter regions of these target genes [21], was only marginally affected by BDSF. The expression and regulation of selected target genes (*bclA*, *cepI*, *aidA*) was validated by quantitative real time PCR using RNA from an independent biological replicate (Table 3). The expression profile of a *cepR* or a *cepI* mutant did not reveal any influence on *rpfF_Bc_* expression by the AHL-dependent QS system, neither at the transcript (Table 3) nor at the protein level [11]. Moreover, *cepR* transcription was not affected by the BDSF signalling molecule. However, qPCR analysis and shotgun proteomics indicated a significant decrease of *cepI* and CepI levels in an *rpfF_Bc_* mutant (Table 3 and Table S2). In agreement with these results we observed that the production of AHL signal molecules was reduced in the *rpfF_Bc_* mutant when compared to the wild type (see below).

**Table 2:** List of genes differentially expressed in the *rpfF_Bc_* and *cepR* mutant, using the wild type as baseline.

Locus ID CCE^a^	Orthologues J2315^b^	Description^c^	Gene name	wt vs *rpfF* d	wt vs *rpfF* comp*l* e	wt vs *cepR*
CCE49231	BCAL0124	Transcriptional activator FlhD		3.7	1.5	0.5
CCE53285	BCAL0831	Phasin family protein		2.8	0.7	3.2
CCE52825	BCAL0833	Acetoacetyl-Coa reductase		4.3	1.1	3.0
CCE51553	BCAL1063	Succinylarginine dihydrolase		2.8	1.4	0.5
CCE50476	BCAL2353	Sulfate permease		18.4	0.9	2.2
CCE51244	BCAL3285	Nitric oxide dioxygenase; flavohemoprotein		6.5	0.6	25.8
CCE46722	BCAM0184	Fucose-Binding lectin protein	*bclB*	181.0	0.5	5.1
CCE46721	BCAM0185	Fucose-Binding lectin protein	*bclC*	73.5	0.7	3.3
CCE46720	BCAM0186	Fucose-Binding lectin protein	*bclA*	274.4	0.5	9.0
CCE46715	BCAM0191	Non-ribosomal peptide synthetase		5.3	0.9	12.8
CCE46713	BCAM0192			9.2	1.5	46.8
CCE46712	BCAM0193			17.1	2.3	47.7
CCE46711	BCAM0194			8.0	1.6	52.2
CCE46710	BCAM0195	Non-ribosomal peptide synthetase		6.5	1.4	34.0
CCE46709	BCAM0196			6.1	0.9	37.7
CCE50917	BCAM0835	Transcriptional regulator, AraC family protein		4.9	1.2	4.3
CCE48735	BCAM1005	Acyltransferase		4.6	0.6	2.2
CCE48728	BCAM1010	Utp–Glucose-1-Phosphate uridylyltransferase		39.4	0.5	2.4
CCE46959	BCAM1745	Atpase		0.01	0.8	0.4
CCE50321	BCAM1871	3-Hydroxy-3-Methylglutaryl-Coenzyme A reductase		3.5	0.8	32.5
CCE53256	BCAM2060	Natural resistance-associated macrophage protein		0.1	0.8	0.4
CCE53120	BCAM2140	Type I secretion membrane fusion protein		16.0	0.9	3.1
CCE53119	BCAM2141	ABC transporter protein		16.0	0.9	4.4
CCE53118	BCAM2142			14.9	1.0	5.3
CCE53117	BCAM2143	Calcium ion binding protein		12.1	0.8	5.1
CCE53089	BCAM2169	Outer membrane autotransporter protein		2.6	0.9	2.1
CCE53021	BCAM2227	Pyochelin biosynthetic protein		na	na	2.2
CCE52940	BCAM2307	Metalloendopeptidase	*zmpB*	3.7	1.4	8.7
CCE52939	BCAM2308	Aminopeptidase		4.0	1.5	5.3
CCE52108	BCAS0292		*aidA'*	3.2	0.9	138.1
CCE52109	BCAS0293		*aidA*	4.0	0.8	167.2

a Nomenclature according to GenBank file CAFQ01000001.1.

b Orthologs were identified as described in the Material and Methods section.

c Description according to EggNOG categories.

d Fold change (FC) of expression, comparing wild type strain with *rpfF* mutant grown in LB medium until an OD of 2.

e Fold change (FC) of expression, comparing wild type strain with complemented *rpfF* mutant grown in LB medium until an OD of 2.

f Fold change (FC) of expression comparing wild type strain with *cepR* mutant grown in LB medium until an OD of 2 [11].

na, not applicable because the read number in the mutant or complemented strains is equal 0.

**Table 3:** Validation of RNA-Seq results using quantitative PCR analysis.

Locus ID CCE^a^	Orthologues J2315^b^	Description^c^	gene name	wt vs *rpfF* d	wt vs *rpfF* compl^e^	wt vs *cepR* f	wt vs *cepR* compl^g^
CCE46720	BCAM0186	Fucose-Binding lectin protein	*bclA*	74	1.2	4.1	0.4
CCE48446	BCAM0581	Enoyl-CoA hydratase/carnithine racemase	*rpfF*	0.6	0.3	0.7	0.8
CCE48447	BCAM0580	EAL/GGDEF domain protein	*rpfR*	1	0.6	0.8	0.5
CCE50322	BCAM1870	N-acyl-L-homoserine lactone synthetase	*cepI*	3.2	1.6	65.0	0.8
CCE50324	BCAM1868	Transcriptional regulator protein	*cepR*	1.7	1.8	0.6	0.3
CCE50476	BCAL2353	Sulfate permease		6	1.8	4.2	1.1
CCE50898	BCAM0854	Mannose-6-phosphate isomerase		11.3	1	6.3	0.6
CCE52109	BCAS0293	AidA	*aidA*	6	1.8	685.0	0.5

a Nomenclature according to GenBank file CAFQ01000001.1.

b Orthologs were identified as described in the Material and Methods section.

c Description according to EggNOG classification.

d Fold change (FC) of expression, comparing wild type strain with *rpfF* mutant grown in LB medium until an OD of 2.

e Fold change (FC) of expression, comparing wild type strain with complemented *rpfF* mutant grown in LB medium until an OD of 2.

f Fold change (FC) of expression comparing wild type strain with *cepR* mutant grown in LB medium until an OD of 2.

g Fold change (FC) of expression, comparing wild type strain with complemented *cepR* mutant grown in LB medium until an OD of 2.

### Both Signal Molecules are Required for Maximal Expression of *bapA* and *bclACB*

In order to understand how the two QS systems co-regulate gene expression, we assessed the impact of AHL and of BDSF on the expression of selected target genes. To this end, we constructed a *cepI rpfF_Bc_* double mutant and used this strain as genetic background to test the effect of the two signal molecules individually and in combination on transcription of target genes. Specifically, we generated *lacZ* transcriptional fusions to the promoter regions of genes that were regulated either by both AHL and BDSF (*bapA*), primarily by BDSF (*bclACB*), or primarly by AHLs (*aidA*) (Table 1, Table 3).

**Figure 2:**
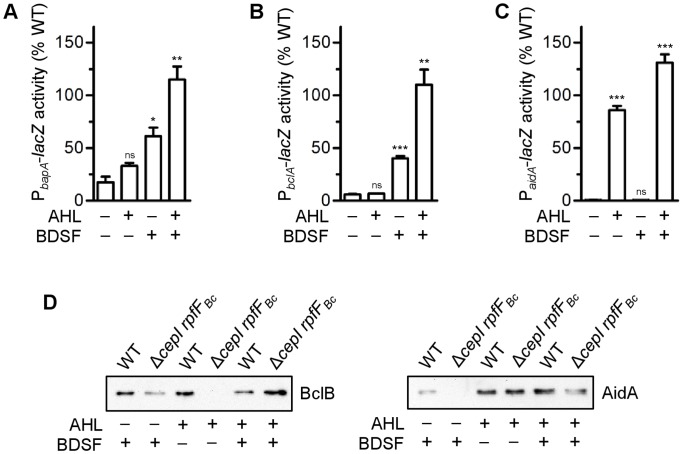
The role of the two QS systems in the regulation of selected genes. The *bapA* (A), *bclA* (B), and *aidA* (C) promoter activities were assessed by means of transcriptional *lacZ* fusions in the H111 wild type strain and in the mutant defective in AHL and BDSF synthesis (Δ*cepI rpfF*_Bc_). The strains were grown to late exponential growth phase in LB Lennox broth in the absence or presence of signal molecules (200 nM C8-HSL; 10 µM BDSF) as indicated by+and - below each bar. Error bars indicate SEM, n = 3. * P<0.05, ** P<0.01, *** P<0.001 (t-test, two-tailed) compared to Δ*cepI rpfF*_Bc_ without signalling molecule (ns, not significant) (D) Expression of BclB and AidA in the H111 wild type and the double mutant Δ*cepI rpfF_Bc_* as assessed by Western Blot analysis. The strains were grown on plates in the presence or absence of signal molecules as indicated by+and - below each band.

In agreement with the global analyses, which showed that *bapA* expression is regulated by both AHLs and BDSF (Table 2), the P*_bapA_*-*lacZ* fusion showed greatly decreased activity in the *cepI rpfF*_Bc_ double mutant compared to the wild type (Figure 2A). Interestingly, the activity of the *bapA* promoter was restored to wild type levels only when the medium was supplemented with both C8-HSL and BDSF (Figure 2A). Addition of either C8-HSL or BDSF resulted in only partial restoration of *bapA* promoter activity. Likewise, activity of the P*_bclA_*-*lacZ* fusion was only restored to the level of the wild type when both signalling molecules were added to the growth medium (Figure 2B). In contrast to the *bapA* promoter the addition of C8-HSL alone did not stimulate transcription of *bclACB* while the addition of BDSF resulted in a partial restoration of *bclACB* promoter activity. To confirm these results we performed a Western Blot analysis using antibodies directed against the lectin BclB (BCAM0184, which is co-transcribed with *blcA* [11]) (Figure 2D). We observed that BclB could only be detected in the double mutant Δ*cepI rpfF*_Bc_ when it was grown in the presence of BDSF. These data further support the idea that expression of BclB is primarily driven by BDSF. We also assessed the promoter activity of *aidA*, a gene already known to be very stringently AHL-regulated [11], [22], in the *cepI rpfF*_Bc_ mutant background. The activity of this promoter fusion was negligible in a double *cepI rpfF*_Bc_ mutant background and could be fully restored after supplementing the medium with AHLs, whereas the addition of BDSF did not show any effect (Figure 2C). In agreement with these results, AidA levels could only be restored after supplementing the strain with AHLs (Figure 2D). In conclusion, our data suggest that the expression of several genes is controlled by both QS systems and that the contribution of each of the systems is variable.

### The BDSF-dependent QS System Affects AHL Levels

**Figure 3:**
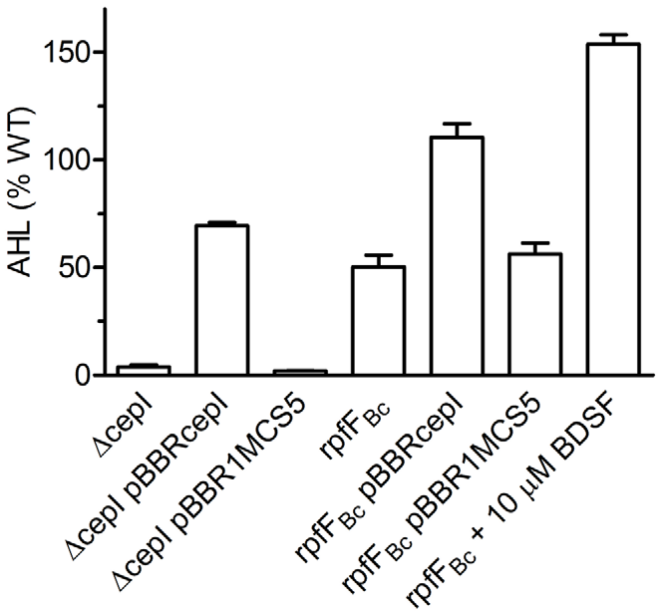
AHL-levels are reduced in an *rpfF_Bc_* mutant. The amount of AHLs produced by the *cepI* and the *rpfF_Bc_* mutant with *cepI* constitutively expressed from plasmid pBBRcepI or with the empty plasmid control pBBR1MCS5 was quantified by the aid of the biosensor *P. putida*/pAS-C8. Error bars indicate SEM, n≥3.

The gene cluster coding for BapA and its export machinery was recently shown to be under the control of CepI/CepR [11]. In the present study we observed that expression of *bapA* in the *rpfF*_Bc_ mutant background was reduced (Table 1, Figure 2A). Given that our data also showed a reduced expression of *cepI* in this mutant background (Table 3, Table S2), we hypothesised that the BDSF-dependent QS system could positively regulate the production of AHLs. To test this hypothesis, we quantified the amount of AHLs produced by the *rpfF*_Bc_ mutant by the aid of the biosensor *P. putida* F117/pAS-C8 [23]. We found that the AHL-levels were 50% lower in the *rpfF*_Bc_ mutant relative to the wild type (Figure 3). The AHL levels were fully rescued when *cepI* was expressed from plasmid pBBRcepI in the BDSF-deficient mutant (Figure 3). These findings were in agreement with our qPCR and proteomics data, which showed a down-regulation of *cepI* in an *rpfF*_Bc_ mutant (Table 3 and Table S2). We also observed that the activity of a P*_cepI_*-*lacZ* promoter fusion was reduced in the *rpfF_Bc_* mutant (Figure S2) and the promoter activity was fully restored after supplementing the medium with either BDSF or C8-HSL. Interestingly, when the fusion was tested in a *cepI* mutant background the promoter activity could not be restored by the addition of BDSF (Figure S2), suggesting that the reduced transcription of *cepI* is a direct consequence of the lowered amounts of AHLs produced by the BDSF mutant.

In agreement with our global analyses, we observed that inactivation of *cepI* or *cepR* did not affect the amount of BDSF produced (Figure S3).

### BDSF and AHLs co-regulate Biofilm Formation and Production of Proteolytic Activity

**Figure 4:**
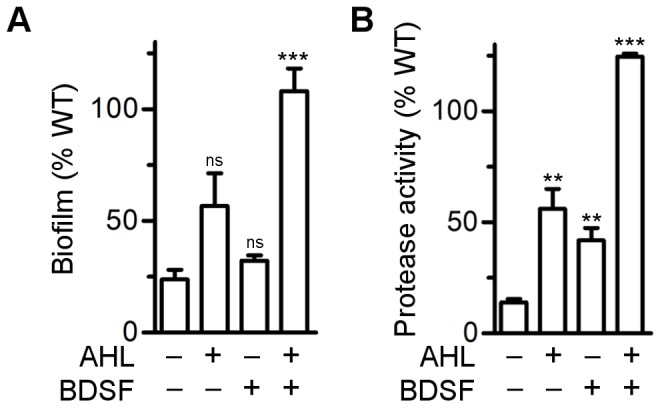
Biofilm formation and protease activity are co-regulated by AHL and BDSF. (A) Biofilm formation in ABC minimal. (B) Protease activity in NYG medium. The strains tested are the wild type H111 and the *cepI rpfF*_Bc_ double mutant. Strains were grown in the presence of absence of signal molecules (200 nM C8-HSL; 10 µM BDSF) as indicated by+and - below each bar. Error bars indicate SEM, n≥3. ** P<0.01, *** P<0.001 (t-test, two-tailed) compared to Δ*cepI rpfF*_Bc_ without signalling molecule (ns, not significant).

We next examined whether the reduced AHL level of the *rpfF*_Bc_ mutant affected known AHL-controlled phenotypes. To this end, we quantified the biofilms formed by the *cepI rpfF*_Bc_ double mutant grown with or without exogenous addition of C8-HSL or BDSF. As shown in Figure 4A, the double mutant was defective in biofilm formation relative to the wild type and this defect could only be restored when both signalling molecules were added to the growth medium. In agreement with this observation, neither overexpression of CepI nor the addition of C8-HSL or BDSF to the growth medium lead to full restoration of the biofilm defect in the *rpfF*_Bc_ mutant (Figure S4).

Previous studies have shown that the proteolytic activity of *B. cenocepacia* H111 is regulated by both AHLs and BDSF [11], [24]. Consistently, we found that transcription of a zinc-metalloprotease (*zmpB*, BCAM2307) was down-regulated in the *rpfF_Bc_* mutant (Table 2). We therefore tested the influence of BDSF and C8-HSL on the production of extracellular proteases of the *cepI rpfF*_Bc_ double mutant. We observed that, similar to biofilm formation, both signalling molecules were needed to fully restore proteolytic activity (Figure 4B).

## Discussion

Our combined RNA-Seq and proteome analysis revealed that the set of genes regulated by BDSF in *B. cenocepacia* H111 shows a substantial overlap with the set of genes recently shown to be CepR-regulated (Figure S1). However, we also identified some genes that were almost exclusively regulated by one of the two QS systems. For example, the gene *aidA*, which encodes a protein required for killing of the nematode *Caenorhabditis elegans* [22], is stringently regulated by C8-HSL (>100-fold at the transcript level, Table 3, Figure 2C and D) whereas the effect of BDSF is marginal (4-fold to 6-fold at the transcript level, Tables 2 and 3, Figure 2C and D). It is important to note that the *aidA* promoter region contains a *cep* box, i.e. a CepR binding site, which is required for AHL-dependent transcriptional activation of this gene [22]. Likewise, we observed that genes containing a *bona fide cep* box in their upstream regions are more strongly affected by the CepI/R than the RpfR/F system (Table S3). At the other extreme, expression of the lectin BclB, which is encoded by the last gene of the *bclACB* operon [11], in the *cepI rpfF_Bc_* double mutant was found to be strongly dependent on BDSF while C8-HSL showed little effect (Figure 2D, Table 2). We have previously shown that in a *cepI* mutant strain transcription of the *bclACB* operon is approximately 6-fold down-regulated relative to the wild type and that this defect is reversed by the addition of C8-HSL to the medium [11]. The evidence presented in this study suggests that AHL-dependent regulation of the *bclACB* operon only occurs when the BDSF-dependent QS system is intact (Figure 2D, Table 3). In summary, these data suggests that the CepIR system synergistically enhances BDSF-dependent activation of *bclACB* expression. The underlying molecular mechanism of how the two QS systems interact in the expression of the lectin operon remains to be elucidated.

It has not escaped our attention that the *rpfF_Bc_* mutant produces significantly reduced amounts of AHL signal molecules, most likely due to lowered transcription of *cepI* (Table 3, Figure S2). This result suggests a hierarchical arrangement of the two QS systems with the RpfF/RpfR system being on top of the CepI/CepR system. However, several of our results do not support this conclusion. Expression of some of the well-characterized AHL-regulated genes, including *aidA*, was only marginally affected in an *rpfF_Bc_* mutant background. In the case of *bapA*, which was shown to be regulated by both systems, addition of AHLs to the BDSF-deficient mutant did not restore expression of this gene to the level of the wild type, which would have been expected if the AHL-dependent circuitry operated downstream of the BDSF system. Likewise, biofilm formation and proteolytic activity was dependent on both signal molecules (Figure 4) and could not be rescued to wild type levels when the BDSF mutant was grown in the presence of AHLs (Figures S4 and S5). Only in the case of proteolytic activity a partial complementation was observed in the presence of AHLs (Figure S5) and this effect may be attributed to the lowered level of the signal molecule. Extracellular proteolytic activity of *B. cenocepacia* is mainly conferred by two metalloproteases, ZmpA and ZmpB [25], [26]. The expression of *zmpA* in a *rpfF_Bc_* mutant of *B. cenocepacia* J2315 was previously shown to be restored when the medium was supplemented with either AHLs or BSDF [27], which may in part explain the slight increase in proteolytic activity when the *cepI rpfF_Bc_* double mutant was grown in the presence of C8-HSL. Importantly, it has been demonstrated that the ZmpB protease has greater activity against casein [26] and thus our measurements mainly reflect the activity of this protease, the expression of which appears to be dependent on both QS systems (Table 2). In conclusion, our data suggest that the reduced AHL levels of the BDSF-deficient mutant are not, at least not solely, responsible for the observed phenotypic defects.

It is noteworthy that the amount of C8-HSL produced by different *B. cenocepacia* strains varies dramatically, with concentrations ranging from 1 nM to 0.2 µM [7]. The CF isolate used in this study, strain H111 [7], [28] produces very high levels (0.2 µM) of C8-HSL. Although the BDSF mutant produces less (50%) AHLs relative to the H111 wild type it still produces much higher amounts of the signal molecule than many other *B. cenocepacia* wild type strains, including the frequently studied strains K56-2 and J2315 [7]. We therefore cannot exclude the possibility that in strains producing very low amounts of C8-HSL inactivation of *rpfF_Bc_* has a more pronounced effect on expression of AHL-regulated functions. In a recent study evidence was presented that inactivation of *rpfF_Bc_* in *B. cenocepacia* J2315 also resulted in a lowered AHL level, albeit this reduction was found to be insignificant when the AHL concentration was normalized against the cell density [27].Given that the amount of AHLs produced by strain J2315 is very low, a further reduction is difficult to quantify with standard methods and this may be the reason for the statistically insignificant results. In addition, the difference between the studies may be due to different growth conditions (Anwar minimal medium versus LB) or the fact that *B. cenocepacia* J2315 harbors an additional QS system [29].

**Figure 5:**
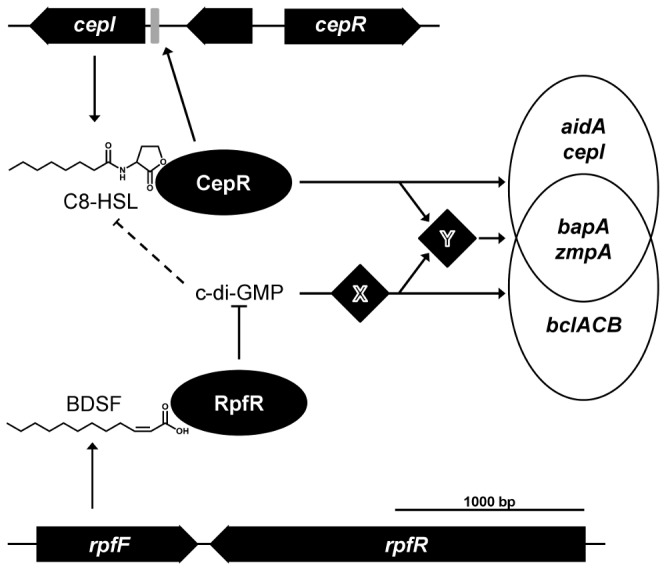
Schematic presentation of the two *B. cenocepacia* H111 QS circuitries. The CepI/CepR system consists of the AHL synthase CepI directing the synthesis of C8-HSL, and of the transcriptional regulator CepR. The RpfF/RpfR system consists of RpfF which directs the synthesis of BDSF, and of its cognate receptor RpfR. Upon binding of BDSF to RpfR the c-di-GMP phosphodiesterase activity of the protein is stimulated and as a consequence the intracellular c-di-GMP level is lowered. The two QS systems operate in parallel to control specific as well as overlapping sets of genes. Our working model assumes an unknown c-di-GMP receptor protein × that stimulates transcription of target genes. Alternatively, the two QS cascades converge and control the expression or the activity status of an unknown common regulator Y, which in turn regulates expression of target genes. C-di-GMP has a negative regulatory effect on AHL levels via an unknown mechanism (depicted by the dashed line).

In conclusion, our data support a model in which the two QS systems operate in parallel to control specific as well as overlapping sets of genes (Figure 5). This model is also in accordance with the finding that the AHL and BDSF stimulons do not completely overlap and some genes are almost exclusively regulated by just one of the QS systems. It has recently been shown that binding of BDSF to its cognate receptor RpfR activates the c-di-GMP phosphodiesterase activity of this protein, which leads to a lowered intracellular c-di-GMP level [16]. At present it is unknown how this change in c-di-GMP level affects transcription of target genes.

In our working model we assume an unknown c-di-GMP receptor protein × that activates transcription of target genes either directly or *via* a regulatory cascade. Given that most genes that were found to be regulated by both QS systems are not directly regulated by CepR/C8-HSL, it is also possible that the two QS cascades converge and control the expression or the activity status of an unknown common regulator Y, which in turn regulates expression of target genes (Figure 5). Work currently under way aims at distinguishing between the two possibilities and at identifying the c-di-GMP effector.

## Materials and Methods

### Bacterial Strains, Plasmids and Growth Conditions

Strains and plasmids used in this study are listed in Table S4. Unless otherwise stated, strains were grown aerobically at 37°C in LB Lennox broth (Difco). Complementation assays were performed with 10 µM BDSF (Sigma) and/or 200 nM C8-HSL (Sigma). Antibiotics were used at the concentrations (in µg/ml) indicated in parentheses: for *E. coli*, ampicillin (100), kanamycin (30), and tetracycline (10); and for *B. cenocepacia*, kanamycin (50), gentamicin (10), trimethoprim (100), and chloramphenicol (60). Growth was monitored spectrophotometrically by measurement of optical density at 600 nm (Ultrospec Pro 2100, GE Healthcare, Switzerland).

### Construction of *B. cenocepacia* H111 Mutants

A deletion mutant of *B. cenocepacia* H111 *cepI* was created using a modified version of the Gateway method described by Choi and Schweizer [30] and modified by Carlier *et al.* [31]. Briefly, the flanking regions of the *cepI* gene were amplified by PCR using oligonucleotide primers cepIDnkan, cepIDnGW, cepIUpGW and cepIUpkan listed in Table S5. A kanamycin-resistance cassette derived from plasmid pKD4 was inserted between the flanking regions of the gene of interest using a PCR overlap technique with primer GW-attB1 and GW-attB2, listed in Table S5. The resulting PCR product containing the Km-resistance cassette flanked by gene-specific DNA was cloned into the Gateway Entry vector pDONR221 using the BP clonase II kit (Invitrogen). The constructs were then genetically transferred into the suicide vector pAUC40 using the LR clonase kit II (Invitrogen), resulting in plasmid pAUC51. The plasmid was introduced into *E. coli* strain S17-1 and conjugally transferred into the wild type strain *B. cenocepacia* H111. Allelic replacement events were selected based on resistance to kanamycin and sensitivity to streptomycin and verified by PCR. Marker-free strain BcepX2 was created by conjugally transferring plasmid pBBR1MCS5::FLP into strain BcepX1. Colonies sensitive to kanamycin were selected and pBBR1MCS-5::FLP was cured after passaging on sucrose-containing medium and selecting colonies sensitive to gentamycin. The loss of the *kanR* cassette was verified by PCR. To generate an *rpfF*_Bc_ insertion mutant, an internal fragment of *rpfF*_Bc_ was amplified by PCR using the primer pair rpfF_Bc_-fw/rpfF_Bc_-rev (Table S5) and inserted as a BamHI/HindIII fragment into the respective sites of plasmid pSHAFT2, generating pSHAFT-rpfF_Bc_. The plasmid was transferred by triparental conjugation to *B. cenocepacia* H111 wild type to generate a BDSF deficient single mutant *rpfF*_Bc_ or to *B. cenocepacia* H111 Δ*cepI* to generate the AHL and BDSF deficient double mutant Δ*cepI rpfF*_Bc_. The integrity of the insertion was verified by PCR using oligonucleotides rpfFBccheck and pSHAFTcheck (Table S5). For constitutive expression of *cepI*, the gene was amplified with primers cepI_orf_fw and cepI_orf_rev (Table S5), digested with *EcoRI* and *XbaI* and cloned into pBBR1MCS-5 cut with the same enzymes.

### RNA Isolation and cDNA Synthesis

Total RNA from wild type and mutant strains grown aerobically in complex LB medium until an OD of 2 was extracted using a modified hot acid phenol protocol [32], further purified (RNeasy kit, Qiagen), and its quality checked using RNA Nano Chips (Agilent 2100 Bioanalyzer; RIN>8). The removal of genomic DNA by DNAseI (Promega) treatment was controlled by a PCR (which targets *aidA*) with 40 cycles. Ten micrograms from each total RNA sample were annealed with random primers (kindly provided by C**.** Majerczyk and P. Greenberg, Seattle) and cDNA synthesized using MLV reverse transcriptase (Promega). Second strand synthesis was carried out according to Yoder-Himes and colleagues [17] using nick translation. Double strand cDNA was purified with MinElute columns (Qiagen).

### Illumina Sequencing and Data Analysis

Libraries were prepared for sequencing according to the manufacturer’s instructions (Illumina). Single-end 51 nucleotide sequence reads were obtained using the Illumina HiSeq2000 system at GATC (Konstanz, Germany), processed with Casava version 1.8 and provided to us in fastq format. Sequencing reads were mapped to the Bcc H111 genome using CLC Genomics Workbench v4.9 (CLCbio) allowing up to 2 mismatches per read. RNA-Seq count data were subsequently analyzed with the DESeq software [18], which ranks the differentially expressed genes according to statistical significance. We chose to rely on the top 150 differentially expressed genes (DESeq p-value <0.1) for our further analyses. The RNA-Seq raw data files are accessible through the GEO Series accession number GSE41244.

### Verification of RNA-Seq Data by Real Time PCR

The expression of H111 orthologs of J2315 genes BCAL2353, BCAM0186, BCAM0580, BCAM0581, BCAM0854, BCAM1868, BCAM1870 and BCAS0293 was analyzed by qPCR using Brilliant III Ultra-Fast SYBR® Green QPCR Master Mix (Agilent, Switzerland) and a Mx3000P instrument (Agilent, Switzerland). As a template, cDNA prepared from biological replicates was used. Each PCR reaction contained 12.5 µl 2× Brilliant III Ultra-Fast SYBR® Green QPCR Master Mix, 0.7 µM of individual primers and 3 dilutions of cDNA in a total volume of 25 µl. Reactions were run in triplicates. Melting curves were generated to verify the specificity of the respective amplification. Gene expression fold-changes were calculated as described elsewhere [33]. Expression of the primary sigma factor *rpoD* (BCAM0918) which, based on the RNA-Seq data, was found to be unchanged under different conditions was used as a reference for normalization. The primers used are listed in Table S5.

### Preparation of Protein Samples for Shotgun Proteomics

Cellular proteins and extracellular proteins were prepared as described previously [34], [35]. Cells were lysed by two consecutive passes through a French Press homogenizer (Hypramag/Aminco). Unbroken cells and cell debris were removed by 15 min centrifugation at 4000 g. Total cell membranes were then harvested by ultracentrifugation for 1 h at 80000 g, 4°C. The pellet containing total membrane proteins was finally dissolved in 100 mM Tris-HCl, pH 7.5, 2% SDS by incubation at 50°C for 1 h. The cell lysate supernatant containing soluble intracellular proteins was extracted with 6 volumes of ice-cold acetone at −20°C overnight. The precipitated proteins were harvested by centrifugation at 20000 g and dissolved in 100 mM Tris-HCl, pH 7.5, 0.1% SDS. Total protein concentration was determined according to Bradford using the Coomassie Plus™ Protein Assay (Pierce) with BSA as a standard. Approximately 15 µg total protein for each extracellular, intracellular and membrane fractions were separated by 1D SDS-PAGE on 12.5% polyacrylamide gels. Gels were stained with colloidal Coomassie Blue (Serva). Individual protein lanes were cut into ten slices and immediately subjected to in-gel tryptic digestion.

### Protein Identification and Further Analysis

Peptides were separated by RP-HPLC and analyzed by a hybrid LTQ-Orbitrap XL mass spectrometer (Thermo Fisher Scientific, Waltham, MA, USA) interfaced with a nanoelectrospray source. Mass spectrometric detection was performed in data-dependent mode. Precursor mass spectra were acquired at the Orbitrap mass analyzer with a scan range from *m/z* 300.0 to 1,600.0 using real-time internal calibration on polydimethylcyclosiloxane (PCM) background ions. Resolution was set to 60,000 at *m/z* 400. Mass spectra processing was performed with Xcalibur 2.0.7 (Thermo Fisher Scientific). Peak list generation for database searches was done with Mascot Distiller 2.1.1.0 (Matrix Science, London, UK). The protein search database was built by combining 7,258 *B. cenocepacia* H111 proteins (downloaded from NCBI link: http://www.ncbi.nlm.nih.gov/nuccore/CAFQ00000000.1) with 260 common contaminants (e.g. human keratin, trypsin). All experimental fragment ion spectra were searched with Mascot 2.3 (Matrix Science, London, UK) against a target and randomized decoy database. The following search parameters were applied: fixed modification, cysteine carbamidomethylation; variable modification, methionine oxidation; enzyme, trypsin; peptide tolerance, ±10 ppm; MS/MS tolerance, ±0.5 Da; maximum number of missed cleavages, 2. PSMs were post-processed with Percolator [36] and stringently filtered such that the final FDR at the PSM level was below 0,1%. All PSMs identified at this stringent FDR were subjected to a PeptideClassifier analysis [37]. Only peptides that unambiguously identify one protein (either class 1a or 3a) were used to generate a minimal list of protein identifications, while shared peptides of class 3b that cannot distinguish between proteins encoded by different gene models were not considered. Furthermore, we did not consider proteins identified by a single spectrum (e.g. those supported by transcriptomics evidence [38]), but required at least 2 independent spectra in order to include true low abundant expressed proteins, while at the same time keeping the FDR rates low without going into manual validation of spectra [39]. Total spectral counts were used for the proteins in each experiment as a basis for DESeq analysis. Due to the lower number of counts compared to sequenced reads, we chose a more lenient cut-off of p<0.2 to select the 116 top-ranked differentially expressed proteins for further analysis. The data associated with this manuscript can be downloaded from the ProteomeCommons.org Tranche network using the following hash: t+/Mpgxtm8V3LTW8MYd5cgth39TXezCbwVchze0oCrAzIF07N18bZSOdSCWF8MhYDqAa27VYeUR+Un7R+kM41GyBUV4AAAAAAABluw =  = .

### Ortholog Mapping and Functional Classification

Throughout the manuscript, we rely on the gene nomenclature of strain J2315 orthologs of H111 genes. Amino acid sequences, including the predicted protein sequences from H111, were clustered using the OrthoMCL v1.4 program [40], with the following parameters: -e 1.0e-6 -v 1000–b 1000, an identity cut-off of 50% and a match cut-off of 50%. We also transferred the functional annotations made by the EggNOG software for the well-studied strain J2315 to H111. The EggNOG resource contains non-supervised orthologous groups that were constructed from 1133 organisms (including J2315 but not H111) [19]. It contains extensive functional annotations, whose depth and coverage differ depending on the evolutionary level that is selected, a feature that is based on the underlying principle of the last common ancestor. Therefore, a greater percentage of annotation is given for more focused groups, e.g. the level proteobacteria (proNOG) has more annotations than the level bacteria. When choosing proteobacteria, we could assign functional predictions to 5270 of 7258 H111 protein coding genes (72.6%).

### Construction and Assessment of Transcriptional *lacZ* Fusions

A modified derivative of pSU11 [41] was constructed by amplifying the *dhfr* cassette from pRN3 with primer pair dhfr-fw/dhfr-rev listed in Table S5 and subsequent cloning of the *sphI* fragment into the respective site of pSU11, giving rise to pSU11Tp. The pP*_bapA_*-*lacZ* promoter-probe vector was then cloned using pSU11Tp as described in [11]. Plasmid pP*_cepI_*-*lacZ* was constructed as pP*_bapA_*-*lacZ* with primer pair PcepI-fw and PcepI-rev. For determination of the ß-galactosidase activity, the strains were grown overnight in LB broth, then subcultured in LB medium complemented with AHL and/or BDSF as indicated and grown to late exponential growth phase. ß-galactosidase quantification was performed as described by Stachel *et al*. [42] with some modifications. Briefly, 50–200 µl of cells were harvested and resuspended in 1 ml Z-buffer. After addition of 25 µl of CHCl_3_ and 25 µl of 0.05% SDS, the cells were vortexed for 10 seconds and then incubated at 30°C for 15 min. The reaction was started by adding 200 µl of ONPG (4 mg/ml) and incubated at 30°C. The reaction was stopped by the addition of 500 µl of 1 M Na_2_CO_3_. Cell debris was removed by centrifugation and absorbance at 442 nm was recorded. β-galactosidase activity was graphed as Miller Units, using the formula Miller Units = (1000*OD420)/(time[min]*V[ml]*OD600). Data are based on three independent biological replicates (n = 3).

### Phenotypic Assays

AHL levels were quantified with *P. putida* F117 pAS-C8 as biosensor. 100 µl of supernatant of an overnight culture was mixed in a black 96-well plate with 100 µl of the biosensor strain grown to exponential growth phase. After 16 hours of incubation at 30°C, fluorescence was recorded using a microtiter plate reader (Synergy HT; Bio-Tek, Germany) with excitation at 485 nm and detection of emission at 528 nm. Background fluorescence (sensor strain with LB medium) was subtracted to give relative fluorescence units (RFU). AHL levels (calculated as RFU/OD_600_) were plotted as percentage of wild type. Data are based on at least 3 independent experiments with 6 technical replicates each.

For quantification of BDSF, one-liter cultures of Bcc strains were grown in YEB medium [43] to an OD_600_ of about 3.5 and centrifuged. The supernatants were acidified to a pH of 4.0 with diluted HCl and extracted with ethyl acetate (1.0, vol/vol) twice. Following evaporation of the ethyl acetate, the residue was dissolved in methanol, subjected to flash chromatography for high-performance liquid chromatography (HPLC) profiling analysis on a reverse-phase column (Phenomenex Luna, 5 M C18, 250 by 4.60 mm) and eluted with 80% methanol in H_2_O at a flow rate of 1 ml min^−1^. Peaks were monitored with a UV detector (210 and 254 nm). Synthetic BDSF was used as control.

Biofilm formation was quantified in a microtiter dish assay as described by Huber *et al*. [44]. Briefly, overnight cultures were diluted to an OD_600_ of 0.01 in AB minimal medium [45] supplemented with 10 mM citrate and 100 µl of this suspension were added per well to a 96-well-plate. After 48 h of static incubation at 30°C, the planktonic cells were removed and 100 µl of a 1% (w/v) aqueous solution of crystal violet were added. Following 20 min incubation at room temperature, the wells were washed thoroughly with distilled water. For quantification, the dye was solubilized by the addition of 120 µl DMSO and absorbance was determined at 570 nm. Biofilm levels were plotted as percentage of the wild type. Data are based on at least 3 independent experiments with 8 technical replicates each.

Proteolytic activity was quantified based on the method described by Safarik *et al*. [46], with some modifications: Bacteria were grown in NYG medium (0.5% peptone, 0.3% yeast extract, 2% glycerol) at 37°C to late exponential growth phase and OD_600_ was recorded. Cells were centrifuged in a microcentrifuge and 100 µl of cell free supernatant was incubated with an equal volume of azocasein (5 mg/ml, in 50 mM Tris-Cl pH 8) for 60 min at 37°C. The proteins were then precipitated by adding 400 µl of 10% TCA in ddH_2_O and removed by centrifugation. The supernatant was incubated with 750 µl of 525 mM NaOH and the absorbance at 442 nm was recorded. Protease activity was calculated as OD_442_/OD_600_ and expressed as percentage of the wild type activity. Data are based on at least 3 independent experiments.

### Western Blot

Bacterial strains were incubated on NB plates (3 g/L Bacto Peptone, 5 g/L meat extract, 1.5% agar), supplemented with 10 µM BDSF or 200 nM C8-HSL or both, for 24 h at 37°C. The cells were scraped of, resuspended in 0.9% NaCl and the OD_600_ was adjusted to 4.0. From this cell suspension, 13 µl were loaded on a 12% SDS-PAGE gel and transferred to a polyvinylidene difluoride (PVDF) membrane (Amersham HybondTM-P, GE Healthcare, Munich, Germany). Membranes were incubated with antibodies against BclB [11] or AidA [22]. Detection was performed as described in Inhülsen *et al*. [11].

## Supporting Information

Figure S1 **Overlap between the RpfF_Bc_ stimulon and CepR regulon.** Venn diagram of the RpfF_Bc_ stimulon (light grey circle) and CepR regulon (dark grey circle) as determined by RNA Seq and microarray analysis, respectively. The number of genes with decreased expression in the *rpfF_Bc_* mutant is shown in brackets.
(TIF)

Figure S2 **Transcription of** ***cepI*** **is reduced in an** ***rpfF_Bc_*** **mutant background.** The activity of a *cepI-lacZ* transcriptional fusion was determined in the wild type, the *rpfF_Bc_* and the *cepI* mutant strain. Exogenous addition of 200 nm C8-HSL (AHL) restored *cepI* promoter activity in both mutant backgrounds, whereas the addition of 10 µM BDSF only rescued activity of the transcriptional fusion in the *rpfF_Bc_* mutant background. Error bars indicate SEM, n = 3.
(TIF)

Figure S3 **BDSF levels are not influenced by the CepI/R system.** BDSF was extracted with ethyl acetate from culture supernatant and quantified by high-performance liquid chromatography (HPLC) as described in the Material and Methods section.
(TIF)

Figure S4 **Neither exogenous addition of AHLs nor** ***in trans*** **expression of** ***cepI*** **rescues the biofilm formation defect of an** ***rpfF_Bc_*** **mutant.** Biofilm formation of the *cepI* and the *rpfF_Bc_* mutant in the presence or absence of 200 nM C8-HSL (AHL) or with *cepI* constitutively expressed from plasmid pBBRcepI (empty plasmid control pBBR1MCS5) using the microtiter plate assay. Error bars indicate SEM, n≥3.
(TIF)

Figure S5 **Biofilm formation and protease activity cannot be rescued to wild type levels when the BDSF mutant is grown in the presence of AHLs.** (A) Biofilm formation and (B) protease activity in the *rpfF_Bc_* mutant. The growth medium was supplemented with 200 nM C8-HSL (AHL), with 10 µM BDSF or both signalling molecules as indicated by+and - below each bar. Error bars indicate SEM, n≥3.
(TIF)

Table S1 **Classification of 150 B. cenocepacia H111 genes that showed differential expression in a** ***rpfF_Bc_*** **mutant strain compared to the wild-type (DE-Seq analysis, p-value <0.1).**
(XLSX)

Table S2 **Classification of 116 B. cenocepacia H111 proteins that showed differential expression in a rpfF mutant strain compared to the wild-type (DE-Seq analysis, p-value <0.2).**
(XLSX)

Table S3 Comparison of AHL and BDSF dependent transcriptional regulation of genes **with an experimentally verified** ***cep*** **box.**
(DOCX)

Table S4 **Bacterial strains and plasmids used in this study.**
(DOCX)

Table S5 **Oligonucleotides used in this study.**
(DOCX)
