# Supplementary figures and images for: The AHL- and BDSF-Dependent Quorum Sensing Systems Control Specific and Overlapping Sets of Genes in Burkholderia cenocepacia H111
Source: PLoS One. 2012 Nov 20;7(11):e49966. doi: 10.1371/journal.pone.0049966 (PMC3502180; doi:10.1371/journal.pone.0049966)

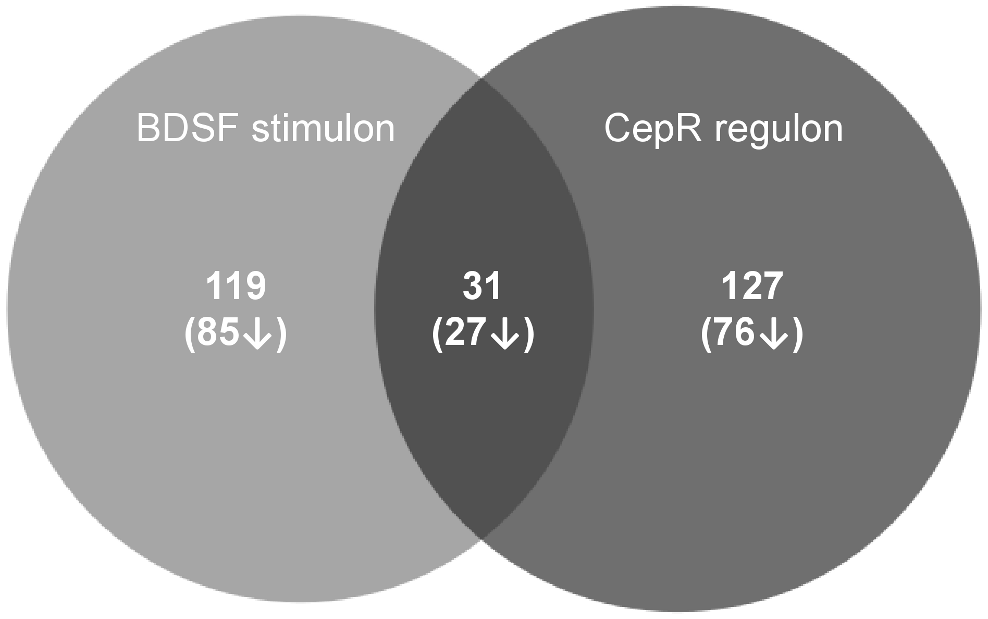

Supplement: Figure S1 — Overlap between the RpfFBc stimulon and CepR regulon. Venn diagram of the RpfFBc stimulon (light grey circle) and CepR regulon (dark grey circle) as determined by RNA Seq and microarray analysis, respectively. The number of genes with decreased expression in the rpfFBc mutant is shown in brackets. (TIF) [file pone.0049966.s001.tif]

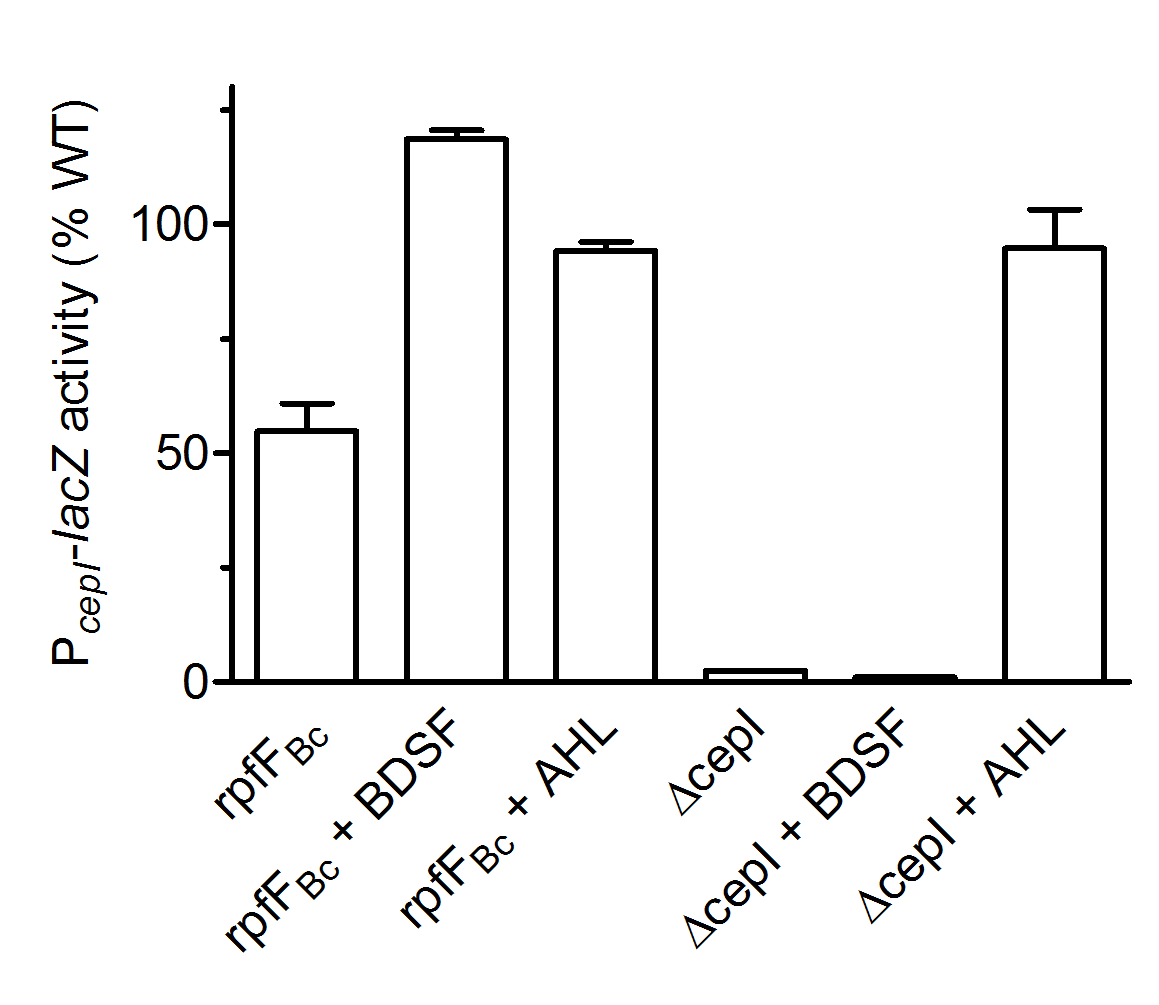

Supplement: Figure S2 — Transcription of cepI is reduced in an rpfFBc mutant background. The activity of a cepI-lacZ transcriptional fusion was determined in the wild type, the rpfFBc and the cepI mutant strain. Exogenous addition of 200 nm C8-HSL (AHL) restored cepI promoter activity in both mutant backgrounds, whereas the addition of 10 µM BDSF only rescued activity of the transcriptional fusion in the rpfFBc mutant background. Error bars indicate SEM, n = 3. (TIF) [file pone.0049966.s002.tif]

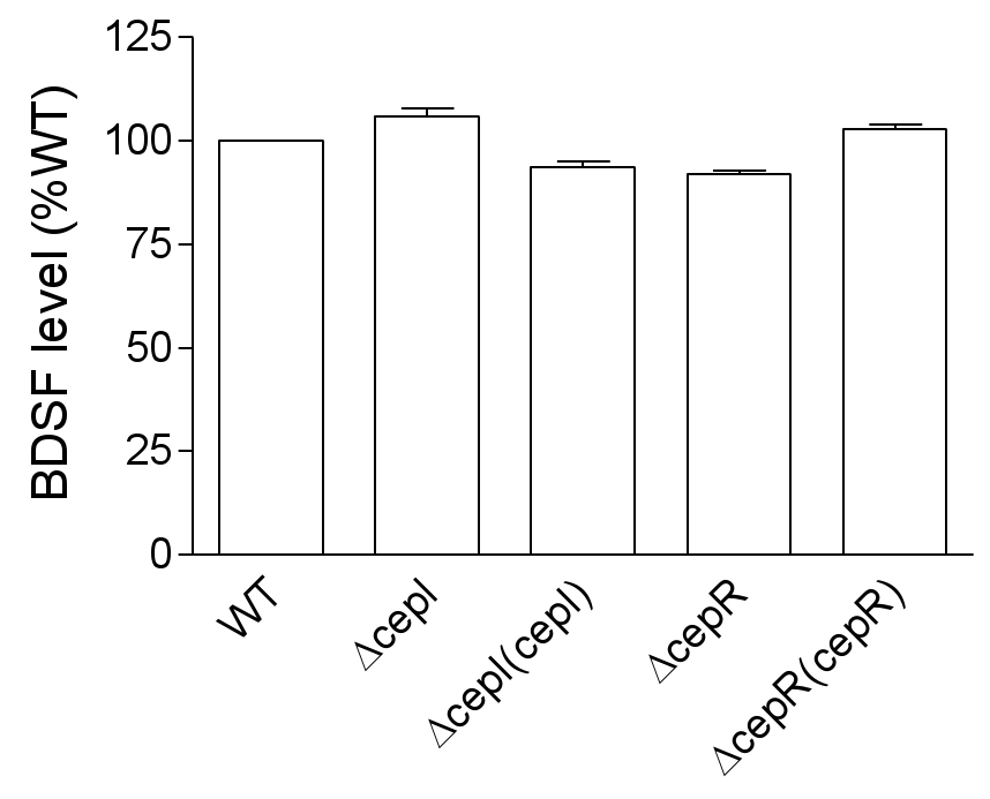

Supplement: Figure S3 — BDSF levels are not influenced by the CepI/R system. BDSF was extracted with ethyl acetate from culture supernatant and quantified by high-performance liquid chromatography (HPLC) as described in the Material and Methods section. (TIF) [file pone.0049966.s003.tif]

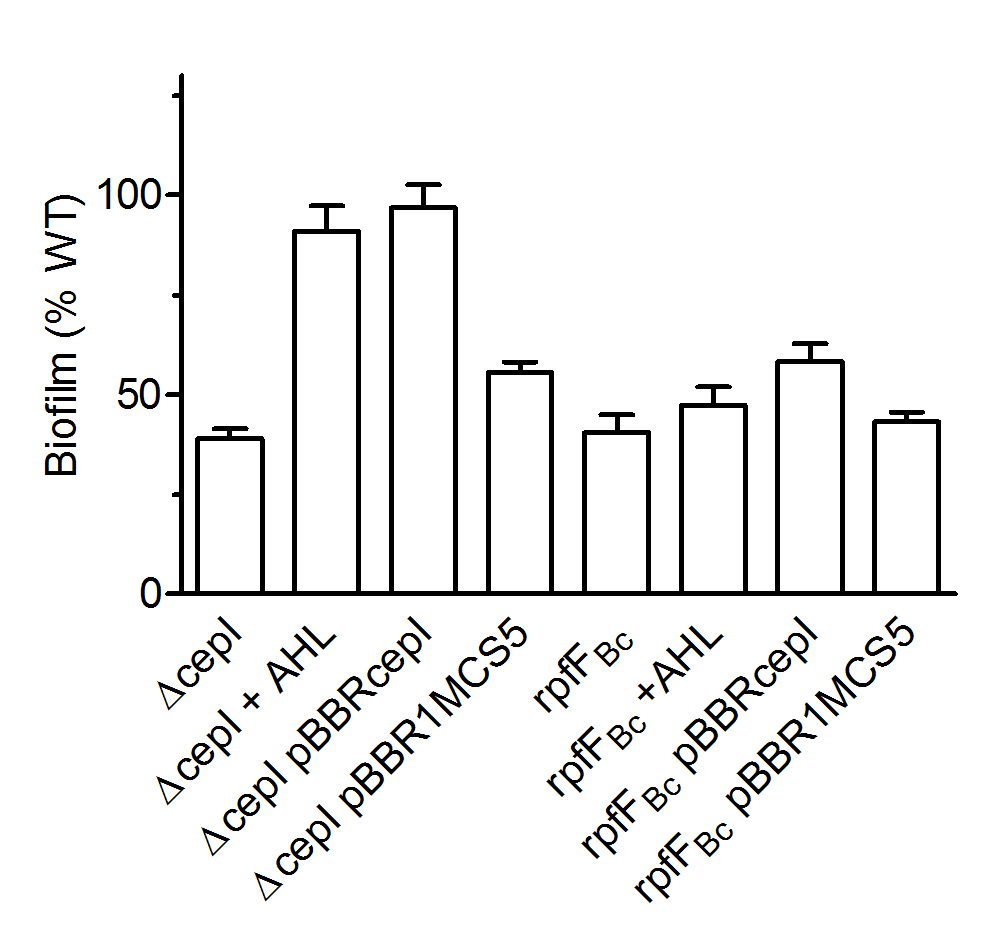

Supplement: Figure S4 — Neither exogenous addition of AHLs nor in trans expression of cepI rescues the biofilm formation defect of an rpfFBc mutant. Biofilm formation of the cepI and the rpfFBc mutant in the presence or absence of 200 nM C8-HSL (AHL) or with cepI constitutively expressed from plasmid pBBRcepI (empty plasmid control pBBR1MCS5) using the microtiter plate assay. Error bars indicate SEM, n≥3. (TIF) [file pone.0049966.s004.tif]

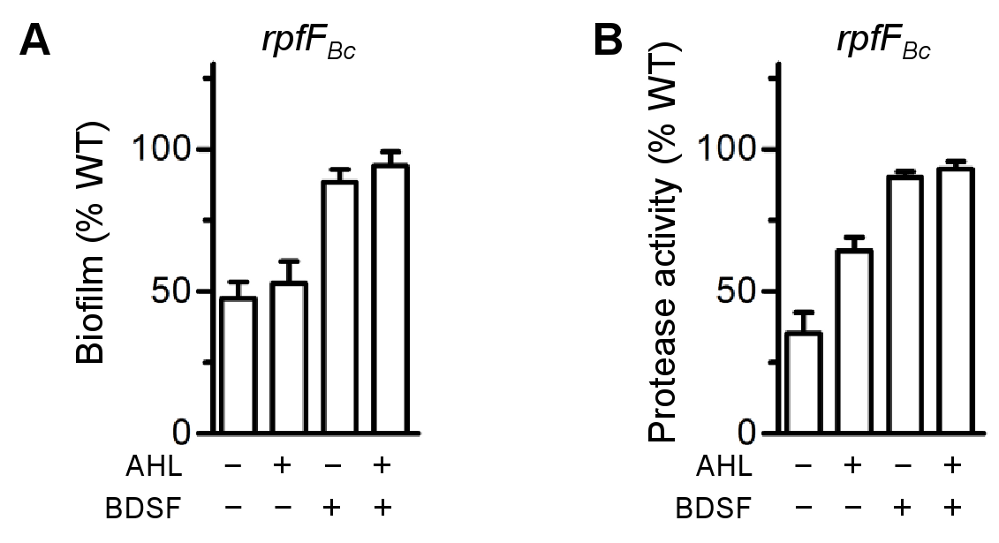

Supplement: Figure S5 — Biofilm formation and protease activity cannot be rescued to wild type levels when the BDSF mutant is grown in the presence of AHLs. (A) Biofilm formation and (B) protease activity in the rpfFBc mutant. The growth medium was supplemented with 200 nM C8-HSL (AHL), with 10 µM BDSF or both signalling molecules as indicated by+and - below each bar. Error bars indicate SEM, n≥3. (TIF) [file pone.0049966.s005.tif]
