# Supplementary material for: The AHL- and BDSF-Dependent Quorum Sensing Systems Control Specific and Overlapping Sets of Genes in Burkholderia cenocepacia H111
Source: PLoS One. 2012 Nov 20;7(11):e49966. doi: 10.1371/journal.pone.0049966 (PMC3502180; doi:10.1371/journal.pone.0049966)
Supplement: Table S3 — Comparison of AHL and BDSF dependent transcriptional regulation of genes with an experimentally verified cep box. (DOCX) [file pone.0049966.s008.docx]

# **Table S3.** Comparison of AHL and BDSF dependent transcriptional regulation of genes with an experimentally verified *cep* box.

| **gene** | ***cepR* vs wild-type** | **wild-type vs *rpfF_Bc_*** |
| --- | --- | --- |
| BCAL0510 | -5.7 | 0.8 |
| BCAM1869 | -6.1 | 0.6 |
| BCAM1870 (*cepI*) | -27 | 1.2 |
| BCAS0156 | not regulated | 3.4 |
| BCAS0293 (*aidA*) | -167 | 4.1 |

# Genes containing a promoter region *cep* box to which CepR was shown to bind directly by Wei et al. [1] are listed in the first column. The second and the third columns display fold change of expression in a *cepR* or a *rpfF_Bc_* mutant, respectively, as assessed by Inhülsen et al. [2] and in this study.

References:

1. Wei Y, Ryan GT, Flores-Mireles AL, Costa ED, Schneider DJ, et al. (2011) Saturation mutagenesis of a CepR binding site as a means to identify new quorum-regulated promoters in *Burkholderia cenocepacia*. Mol Microbiol 79: 616–632. doi:10.1111/j.1365-2958.2010.07469.x.

2. Inhülsen S, Aguilar C, Schmid N, Suppiger A, Riedel K, et al. (2012) Identification of functions linking quorum sensing with biofilm formation in *Burkholderia cenocepacia* H111. MicrobiologyOpen 1: 225–242. doi:10.1002/mbo3.24.
