# Supplementary material for: The AHL- and BDSF-Dependent Quorum Sensing Systems Control Specific and Overlapping Sets of Genes in Burkholderia cenocepacia H111
Source: PLoS One. 2012 Nov 20;7(11):e49966. doi: 10.1371/journal.pone.0049966 (PMC3502180; doi:10.1371/journal.pone.0049966)
Supplement: Table S4 — Bacterial strains and plasmids used in this study. (DOCX) [file pone.0049966.s009.docx]

# **Table S4:** Bacterial strains and plasmids used in this study.

| **Strain or plasmid** | **Description** | **Reference** |
| --- | --- | --- |
| Strains |  |  |
| *E. coli* |  |  |
| DH5α | F^-^ Φ80*lacZ*∆M15 ∆(*lacZYA-argF*) *recA1 endA gyrA96 thi-1 hsdR17 supE44 relAl deoR(U169)* | [1] |
| MM294 | F^-^ *endA1 hsdR17 supE44*(AS*) rfbD1 spoT1 thi-1* | [2] |
| S17-1 | RP4 Mob+ | [3] |
| DB3.1λpir | λpir lysogen of strain DB3.1 | [4] |
| Top10 | Δ*lacX74 ara*Δ*139*Δ*(ara-leu)* | Invitrogen |
| *B. cenocepacia* |  |  |
| H111 | CF isolate from Germany, genomovar III | [5,6] |
| H111-R | *cepR*::Km mutant of H111, Km^R^ | [7] |
| H111 ΔcepI | Δ*cepI* mutant of H111, markerless | This study |
| H111-rpfF_Bc_ | *rpfF_Bc_*::pSHAFT2 mutant of H111, Cm^R^ | This study |
| H111 ΔcepI rpfF_Bc_ | Δ*cepI* and rpfF_Bc_::pSHAFT double mutant, Cm^R^ | This study |
| Plasmids |  |  |
| pP_aidA_-*lacZ* | pSU11 containing the putative *aidA* promoter region | [8] |
| pAUC40 | suicide vector, Gateway attR-CmR cassette cloned in pKNG101, *Sm^R^*, *Cm^R^* | [9] |
| pAUC51 | *cepI::Km^R^* cloned in pAUC40 | This study |
| pP*_bapA_-lacZ* | pSU11Tp containing the putative *bapA* promoter region | [8] |
| pBBR1MCS | broad host-range cloning vector; Cm^R^ | [10] |
| pBBR1MCS-5 | broad host-range cloning vector; Gm^R^ | [11] |
| pBBR1MCS5::FLP | Derivative of pBBR1MCS-5 harboring the FLP and *sacB* cassette from pFLP2, *Gm^R^* | This study |
| pBBRcepI | pBBR1MCS-5 containing the *cepI* gene of *B. cenocepacia* H111, Gm^R^ | This study |
| pBBR-cepR | pBBR1MCS containing the *cepR* gene of *B. cenocepacia* H111; Cm^R^ | [8] |
| pP*_bclA_*-*lacZ* | pSU11 containing the *bclA* promoter region | [8] |
| pP*_cepI_*-*lacZ* | pSU11 containing the *cepI* promoter region | This study |
| pDONR221 | Gateway donor plasmid, *Kan^R^* | Invitrogen |
| pFLP2 | Plasmid source of the FLP cassette | [12] |
| pKD4 | source of *KanR* cassette | [12] |
| pRK2013 | RK2 derivative, *mob^+^ tra*^+^ *ori* ColE1; Km^R^ | [13] |
| pRN3 | source of dhfr cassette | [14] |
| pSHAFT2 | Broad-host-range suicide plasmid, mobilisable for conjugation, Cm^R^ | S. Shastri / M.S. Thomas, manuscript in preparation |
| pSHAFT-rpfF_BC_ | pSHAFT2 containing an internal fragment of *rpfF_Bc_* | This study |
| pSU11 | promoter probe vector; Gm^R^ | [14] |
| pSU11Tp | pSU11 derivative harboring dhfr cassette from pRN3, Tp^R^ | This study |

References:

1. Hanahan D (1983) Studies on transformation of *Escherichia coli* with plasmids. Journal of Molecular Biology 166: 557–580. doi:10.1016/S0022-2836(83)80284-8.

2. Meselson M, Yuan R (1968) DNA restriction enzyme from *E. coli*. Nature 217: 1110–1114. doi:10.1038/2171110a0.

3. Simon R, Priefer U, Pühler A (1983) A Broad Host Range Mobilization System for In Vivo Genetic Engineering: Transposon Mutagenesis in Gram Negative Bacteria. Bio/Technology 1: 784–791. doi:10.1038/nbt1183-784.

4. House BL, Mortimer MW, Kahn ML (2004) New Recombination Methods for *Sinorhizobium meliloti* Genetics. Applied and Environmental Microbiology 70: 2806–2815. doi:10.1128/AEM.70.5.2806-2815.2004.

5. Römling U, Wingender J, Müller H, Tümmler B (1994) A major *Pseudomonas aeruginosa* clone common to patients and aquatic habitats. Appl Environ Microbiol 60: 1734–1738.

6. Gotschlich A, Huber B, Geisenberger O, Tögl A, Steidle A, et al. (2001) Synthesis of multiple *N*-acylhomoserine lactones is wide-spread among the members of the *Burkholderia cepacia* complex. Syst Appl Microbiol 24: 1–14. doi:10.1078/0723-2020-00013.

7. Huber B, Riedel K, Hentzer M, Heydorn A, Gotschlich A, et al. (2001) The *cep* quorum-sensing system of *Burkholderia cepacia* H111 controls biofilm formation and swarming motility. Microbiology 147: 2517–2528.

8. Inhülsen S, Aguilar C, Schmid N, Suppiger A, Riedel K, et al. (2012) Identification of functions linking quorum sensing with biofilm formation in *Burkholderia cenocepacia* H111. MicrobiologyOpen 1: 225–242. doi:10.1002/mbo3.24.

9. Carlier A, Burbank L, von Bodman SB (2009) Identification and characterization of three novel EsaI/EsaR quorum-sensing controlled stewartan exopolysaccharide biosynthetic genes in *Pantoea stewartii* ssp. *stewartii*. Mol Microbiol 74: 903–913. doi:10.1111/j.1365-2958.2009.06906.x.

10. Kovach ME, Phillips RW, Elzer PH, Roop RM, Peterson KM (1994) pBBR1MCS: a broad-host-range cloning vector. Biotechniques 16: 800–802. doi:51199426.

11. Kovach ME, Elzer PH, Steven Hill D, Robertson GT, Farris M a, et al. (1995) Four new derivatives of the broad-host-range cloning vector pBBR1MCS, carrying different antibiotic-resistance cassettes. Gene 166: 175–176. doi:10.1016/0378-1119(95)00584-1.

12. Datsenko KA, Wanner BL (2000) One-step inactivation of chromosomal genes in *Escherichia coli* K-12 using PCR products. Proc Natl Acad Sci U S A 97: 6640–6645. doi:10.1073/pnas.120163297.

13. Figurski DH, Helinski DR (1979) Replication of an origin-containing derivative of plasmid RK2 dependent on a plasmid function provided in trans. Proc Natl Acad Sci U S A 76: 1648–1652.

14. O’Grady EP, Viteri DF, Malott RJ, Sokol PA (2009) Reciprocal regulation by the CepIR and CciIR quorum sensing systems in *Burkholderia cenocepacia*. BMC Genomics 10: 441. doi:10.1186/1471-2164-10-441.
