# Supplementary material for: The AHL- and BDSF-Dependent Quorum Sensing Systems Control Specific and Overlapping Sets of Genes in Burkholderia cenocepacia H111
Source: PLoS One. 2012 Nov 20;7(11):e49966. doi: 10.1371/journal.pone.0049966 (PMC3502180; doi:10.1371/journal.pone.0049966)
Supplement: Table S5 — Oligonucleotides used in this study. (DOCX) [file pone.0049966.s010.docx]

# **Table S5.** Oligonucleotides used in this study.

| **Primer** | **Sequence (5' - 3')^a^** |
| --- | --- |
| cepI_orf_fw | CCGAATTCAGCACAGATCCGAGGACAT |
| cepI_orf_rev | CCTCTAGAAGACTGGTCCACGTTGGTT |
| cepIDnGW | TACAAGAAAGCTGGGTACGCGTGCCACAGCAATTCG |
| cepIDnkan | CGGAATAGGAACTAAGGAGGATATTCATATGCTGGTGGTCGCGTGCTGGAT |
| cepIUpGW | TACAAAAAAGCAGGCTCCGGCTATACCGAATGGCATCG |
| cepIUpkan | GAACTTCGAAGCAGCTCCAGCCTACGCCCTTCCTCGTGAACGAA |
| dhfR-fw | GCATGCGGTCTGACGCTCAGTGGAACG |
| dhfR-rev | GCATGCGCTTAGGCCACACGTTCAAG |
| GW-attB1 | GGGGACAAGTTTGTACAAAAAAGCAGGCT |
| GW-attB2 | GGGGACCACTTTGTACAAGAAAGCTGGGT |
| Pbap-fw | CTCGAGCGAATAAGGTGAACCAATCCAG |
| Pbap-rev | AAGCTTATAGTTGGCCACGTCCTCTTT |
| PcepI-fw | GGCTCGAGGGCCGCGCATTCCTCTGACG |
| PcepI-rev | GGAAGCTTGCCGATAGCGCCCGAGATCC |
| pKD4fwd | TAGGCTGGAGCTGCTTCGAAGTTC |
| pKD4rev-2 | CATATGAATATCCTCCTTAGTTCCTATTCCG |
| pSHAFTcheck | AAGGTGACCGCGTATTATTA |
| rpfF_Bc_check | AAGCTTAACCACATCTCACGAGGACA |
| rpfF_Bc_-fw | AAGCTTCCGCAGTCTTCGTACCACTC |
| rpfF_Bc_-rev | GGATCCTTCAACCAGCAACTCGTCAC |

^a^ restriction sites used for cloning are underlined
